# Optimizing lifestyle profiles is potential for preventing nonalcoholic fatty liver disease and enhancing its survival

**DOI:** 10.1038/s41598-024-55566-9

**Published:** 2024-03-06

**Authors:** Beilin Tu, Wei Li, Haitao Xiao, Xuewen Xu, Yange Zhang

**Affiliations:** 1 Department of Plastic and Burns Surgery, West China Hospital, Sichuan University, 37 Guoxue Road, Chengdu, 610041 China; 2 Department of Liver Surgery and Liver Transplantation, State Key Laboratory of Biotherapy and Cancer Center, West China Hospital, Sichuan University and Collaborative Innovation Center of Biotherapy, Chengdu, 610041 China

**Keywords:** Non-alcoholic fatty liver disease, Latent profile analysis, Lifestyle, Survival, Diseases, Non-alcoholic fatty liver disease

## Abstract

The aim of this study was to evaluate the association between lifestyle profile and disease incidence/mortality in patients with non-alcoholic fatty liver disease (NAFLD). Lifestyle profiles ascertainment was based on the latent profile analysis. The associations of lifestyle profile and outcomes were analyzed by multivariate logistic or Cox regressions. Four lifestyle profiles (profile 1 and 2 for male, profile 3 and 4 for female) were established for all participants. Compared to profile 1, profile 2 (*P* = 0.042) and profile 3 (*P* = 0.013) had lower incidence for NAFLD. In contrast, profile 4 showed similar NAFLD prevalence compared to profile 1 (*P* = 0.756). Individuals with NAFLD within profile 3 had the best long-term survival, and the HR was 0.55 (95% CI 0.40–0.76) for all-cause mortality (compared to profile 1). Profile 4 (*P* = 0.098) and profile 2 (*P* = 0.546) had similar all-cause survival compared to profile 1. We explored the associations of healthy lifestyle score with mortality and incidence of NAFLD stratified by lifestyle profiles. We observed that with the increase of healthy lifestyle score, participants within profile 2 did not display lower NAFLD incidence and better long-term survival in NAFLD cases. In this study, lifestyle profiles were constructed in NHANES participants. The distinct lifestyle profiles may help optimize decision-making regarding lifestyle management in preventing NAFLD development, as well as selection of a more personalized approach for improving NAFLD survival.

## Introduction

Non-alcoholic fatty liver disease (NAFLD), the most prevalent liver disease worldwide, is identified as the hepatic expression of metabolic syndrome^1^. NAFLD is significant related to morbidity and mortality caused by cirrhosis, hepatocellular carcinoma, diabetes or cardiovascular disease^2,3^. The etiology of NAFLD reflects multiple interactions between environmental and genetic risk factors^4^. In general, the development of NAFLD is closely associated with lifestyle factors, namely the excessive intake of calorie-dense food as well as decreased physical activity and exercise^5,6^. Consequently, lifestyle modification such as dietary strategies and exercise training in the treatment of NAFLD was proved to be significant in inducing improvement and/or even remission of NAFLD^7–12^.

The associations of multiple lifestyle factors including smoking, diet and physical activity (PA) and NAFLD incidence were analyzed in previous studies, and statistical significances were observed in these reports^13–15^. However, to our knowledge, no attention has been paid to the role of overall lifestyle behaviors in the development and mortality of NAFLD. Exploring whole lifestyle patterns, rather than the individual components, has become increasingly significant in determining lifestyle and disease relations^16–18^. Given the complex interaction and correlation between different lifestyles, lifestyle pattern analysis has emerged as a more comprehensive evaluation method for overall lifestyle assessment. In this study, using latent profile analysis (LPA)^18^, we examined the association between lifestyle patterns, characterized by lifestyle profiles, and the risk of NAFLD development and all-cause death in cases with NAFLD. The lifestyle risk factors for outcomes included diet quality, total physical activity, leisure time physical activity, smoking status, alcohol consumption (according to NAFLD definition, significant alcohol users were excluded), sedentary time and sleep hour.

## Methods

### Study population

Participants were drawn from the National Health and Nutrition Examination Survey (NHANES) 2007–2014. NHANES is a continuous survey compiled in 2-year cycles by the National Center for Health Statistics (NCHS) of the United States Centers for Disease Control and Prevention (CDC). NHANES is a nationally representative sample of the civilian, non-institutionalized US population. Details of the methods and procedures such as survey design utilized in NHANES were described in the NHANES website (https://www.cdc.gov/nchs/nhanes/). The current study was restricted to participants, ages > 18 years, with available data on lifestyle factors (diet, total physical activity, leisure time physical activity, smoking status, alcohol consumption, sedentary time and sleep hour), covariates (sex, age, race, education, marital status, family income to poverty ratio, employment, insurance), and outcomes (hepatic steatosis index and survival time/survival status) (Fig. S1). The study protocol conformed to the ethical standards of the 1964 declaration of Helsinki and its later amendments. All procedures involving human participants were approved by the National Center for Health Statistics Research Ethics Review Committee, and all participants signed informed consent forms. All participant records were anonymised before being accessed by the authors.

### Definition of NAFLD and mortality

According to previous studies^19,20^, we defied NAFLD based on the hepatic steatosis index (HSI). HSI was computed using aspartate aminotransferase (AST), alanine aminotransferase (ALT), body mass index (BMI) and diabetes. It was calculated according to the following formula: HSI = 8 × (ALT/AST ratio) + BMI (+ 2, if female; + 2, if diabetes). HSI score > 36 was defined as presence of NAFLD, and HSI < 30 was considered as non-NAFLD. The mortality outcome of participants with NAFLD was determined by the National Death Index (NDI) records. Follow-up time was calculated as the time (in months) from NHANES interview date until the date of death from any cause or end of follow-up on 31 December 2015.

### Lifestyle ascertainment and the other covariates

Levels of physical activity was self-reported by participants through the physical activity questionnaire. The PA questionnaire gathered data on work and recreational activities. The number of days and the minutes of PA were collected simultaneously. For data from NHANES 2007–2014, the Metabolic Equivalent of Task (MET)-minutes per week could be computed by multiplying the total number of minutes per week and the respective MET level of each activity (vigorous work/recreational-related activity = 8 MET, moderate work/recreational-related activity = 4 MET)^21^. The total MET-minutes per week comprised the sum of both work and recreational-related activity. The leisure-time physical activity (LTPA) was represented only by the MET-minutes per week of the recreational-related activity. Sedentary time was coded as daily hours, and was calculated by summing the time of sitting or reclining at work, at home, or at school, including time spent sitting at a desk, sitting with friends, traveling in a bus, car, or train, reading, playing cards, watching television, or using a computer. Diet quality was assessed by the Healthy Eating Index (HEI) score^22^. The total nutrient intakes (DR1TOT and DR2TOT) were used to calculate scores of the 13 components of HEI-2015. A higher total score corresponds to a healthier diet. The alcohol use questionnaire in NHANES was designed to collect data related to the frequency and quantity of alcohol consumption. Alcohol drink was defined as the average number of drinks per day over a period of 12 months. The definition of a drink was an ounce of liquor, a 5-oz glass of wine, or a 12-oz beer^19^. The intensity of smoking was expressed as the number of cigarettes smoked per day for current or ever smokers. Individuals without smoking in their entire life was recorded as 0 cigarette. Usual weekday or workday sleep hour was self-reported by participants. A healthy lifestyle was defined by time of LTPA and total PA above median, sedentary hours below median, HEI score above median, alcohol intake below median, cigarettes smoked per day below median and sleep hour between 6 and 8 h. The summing of the number of healthy lifestyles was defined as the healthy lifestyle score (HLS).

The other self-reported covariates by the participants included: age (continuous), sex (male or female), race (non-Hispanic White; non-Hispanic Black; Mexican American; the other), marital status (married/cohabited; widowed; divorced/separated; unmarried), education level (less than 9th grade; 9-12th grade or equivalent; college or above) employment status (employed or unemployed), insurance (insured or uninsured) and family income-to-poverty ratio. Comorbidities of participants were self-reported as yes or no in questionaries including hypertension, diabetes, cancer, cardiovascular disease (CVD), stroke and chronic obstructive pulmonary disease (COPD) such as emphysema/chronic bronchitis. Laboratory indicators included high-density lipoprotein cholesterol, total cholesterol and fasting triglycerides. Fibrosis-4 (FIB-4) score was calculated with the formula: Age (years) * AST (IU/L)/Platelet count (10^9^/L) * ALT (IU/L)^1/2^^23^.

### Statistical analysis

Participants’ characteristics, stratified by lifestyle profiles, were presented as mean ± standard deviation (SD) or median (min–max) for continuous variables, and as frequency (%) for categorical or ordinal variables. Given the complex survey design of the NHANES, we utilized appropriate sample weights, stratification, and clustering to ensure the data representative for the entire US populations (using the ‘survey’ package). Logistic regression models were applied to determine the associations of lifestyle profiles and NAFLD development. Multivariate Cox regressions were used to examine the associations of lifestyle profiles and participant survival. All models were adjusted for confounders considered a priori to be associated with NAFLD development and prognosis.

LPA was a Gaussian finite mixture modeling method utilized to identify distinct clusters^24^. In this study, LPA (analyzed by the ‘tidyLPA’ package) was used to identify the underlying lifestyle profiles based on all seven continuous lifestyle components. All of the seven factors were scaled by the Z-scores before LPA. The distributions of included variables were examined before analysis, and severely skewed data was transformed. Several statistical fit indices were utilized to evaluate model fit and to determine the optimal number of unique profiles: Bayesian information criteria (BIC), Akaike information criterion (AIC), consistent Akaike information criterion (CAIC), sample-size adjusted Bayesian Information Criterion (SABIC) and the entropy. *P* values < 0.05 were considered statistically significant. Statistical analyses were performed using R software, version 4.1.1.

### Ethics approval and consent to participate

The protocol of NHANES was approved by the institutional review board of the National Center for Health Statistics, CDC. Written informed consent was obtained from all participants before participation in this study.

## Results

### Identify the number of latent profiles

Among 40,617 participants in NHANES 2007–2014, 14,622 cases with (n = 8132) or without (n = 6490) HSI-NAFLD after exclusion were eligible for the analysis. Given the general differences between male and female in lifestyles, LPA analyses were conducted in male and female separately. As shown in Table S1, models with different number of profiles were compared. In both of the male and female analytic subgroups, the entropy dropped remarkably from 2- to 3-profile model. In addition, the BIC and AIC values remained stable among different groups with 2-, 3- or 4-profiles. Consequently, the 2-profile model was chosen as the final one. Table S1 also showed the number of individuals in each profile.

### Different characteristics between profiles

**Figure 1:**
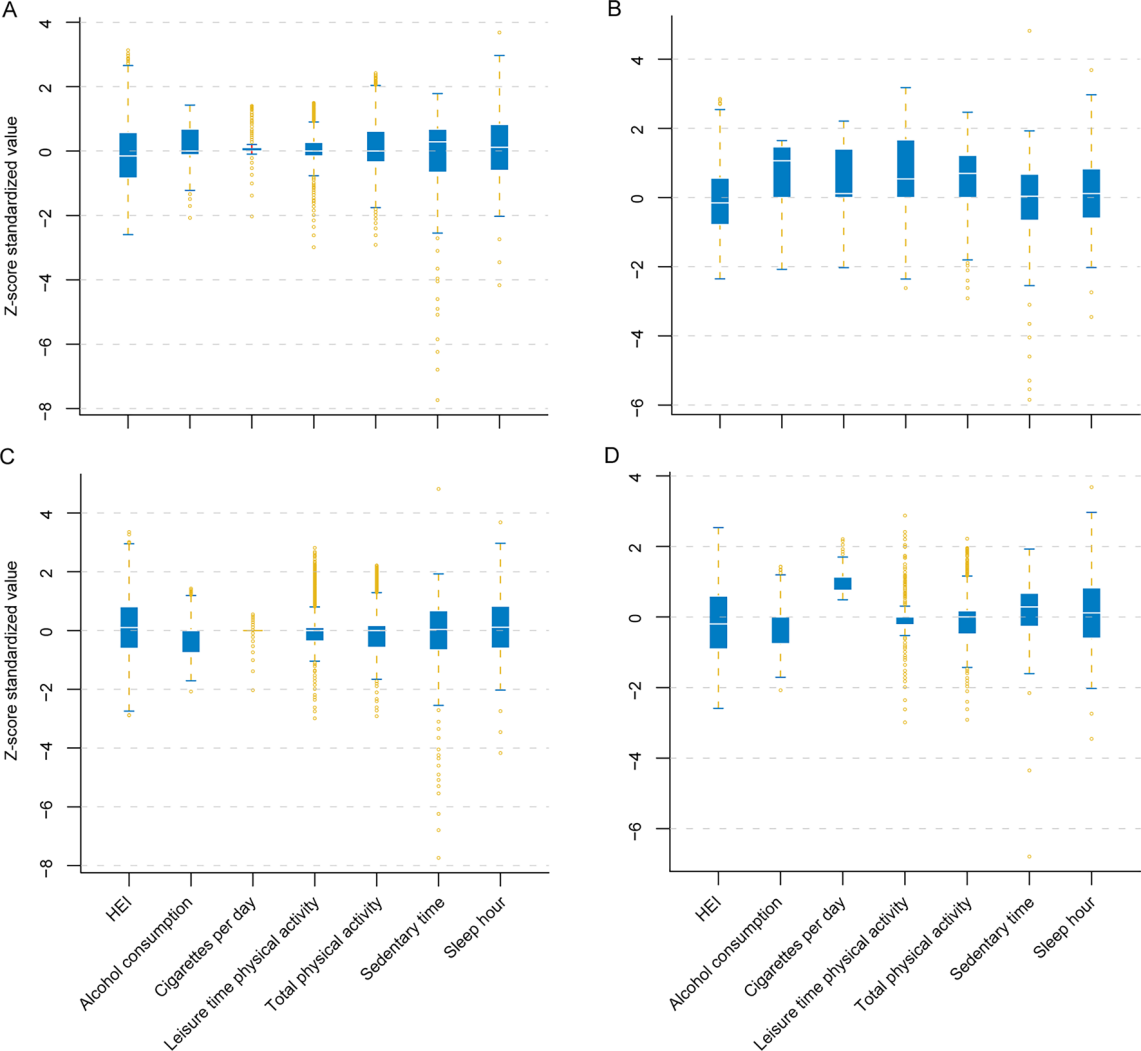
Boxplots are represented for each latent profiles to illustrate Z-score distribution of Healthy Eating Index (HEI) score, alcohol consumption, cigarettes smoking, leisure time physical activity, total physical activity, sedentary time and sleep hour.

In analysis described above, four LPA profiles (two for male; two for female) were finally established. Significant differences in age, race, marital status, education, employment, insurance, family income-to-poverty ratio, laboratory examinations, and comorbidities were observed across LPA profiles (Table 1). Statistically significant differences were found when comparing lifestyle features among four profiles, as summarized in Fig. 1 and Table 1. Profile 2 was characterized by the highest total and leisure PA, but also higher cigarettes and alcohol consumptions. Profile 3 was characterized by the highest HEI score, and lower cigarettes and alcohol consumptions. Profile 4 had the highest cigarettes smoking and lowest total and leisure PA time, and the other lifestyle factors were also unhealthier in this profile. In contrast, lifestyle indicators in profile 1 were moderate compared to the other profiles.

**Table 1:** Baseline characteristics of participants with NAFLD diagnosed by Hepatic Steatosis Index.

Variables	Profile 1 (n = 5951)	Profile 2 (n = 1195)	Profile 3 (n = 6666)	Profile 4 (n = 810)	P value
Age, years	48.9 ± 17.9	48.4 ± 18.5	48.5 ± 17.8	55.3 ± 15.5	< 0.001
Sex					< 0.001
Male	5951 (100.0%)	1195 (100.0%)	0 (0.0%)	0 (0.0%)	
Female	0 (0.0%)	0 (0.0%)	6666 (100.0%)	810 (100.0%)	
Race					< 0.001
Non-Hispanic White	2888 (48.5%)	672 (56.2%)	3020 (45.3%)	613 (75.7%)	
Non-Hispanic Black	1084 (18.2%)	214 (17.9%)	1320 (19.8%)	98 (12.1%)	
Mexican American	937 (15.7%)	151 (12.6%)	1069 (16.0%)	39 (4.8%)	
The other	1042 (17.5%)	158 (13.2%)	1257 (18.9%)	60 (7.4%)	
Marital status					< 0.001
Married/cohabited	4060 (68.2%)	680 (56.9%)	3645 (54.7%)	421 (52.0%)	
Widowed	225 (3.8%)	49 (4.1%)	720 (10.8%)	125 (15.4%)	
Divorced/separated	606 (10.2%)	169 (14.1%)	1062 (15.9%)	188 (23.2%)	
Unmarried	1060 (17.8%)	297 (24.9%)	1239 (18.6%)	76 (9.4%)	
Education					< 0.001
Less than 9th grade	606 (10.2%)	103 (8.6%)	565 (8.5%)	59 (7.3%)	
9–12th grade or equivalent	2194 (36.9%)	469 (39.2%)	2304 (34.6%)	363 (44.8%)	
College or above	3151 (52.9%)	623 (52.1%)	3797 (57.0%)	388 (47.9%)	
Employment					< 0.001
Employed	3758 (63.1%)	691 (57.8%)	3589 (53.8%)	295 (36.4%)	
Unemployed	2193 (36.9%)	504 (42.2%)	3077 (46.2%)	515 (63.6%)	
Family income-to-poverty ratio	2.7 ± 1.6	2.6 ± 1.7	2.5 ± 1.6	2.3 ± 1.6	< 0.001
Insurance					< 0.001
Insured	4484 (75.3%)	889 (74.4%)	5281 (79.2%)	662 (81.7%)	
Uninsured	1467 (24.7%)	306 (25.6%)	1385 (20.8%)	148 (18.3%)	
BMI, kg/m^2^	28.9 ± 6.1	28.7 ± 6.1	29.5 ± 7.5	30.5 ± 7.8	< 0.001
Alanine aminotransferase (IU/L)	28.6 ± 22.3	28.6 ± 16.9	21.2 ± 12.6	21.8 ± 15.5	< 0.001
Aspartate aminotransferase (IU/L)	26.7 ± 17.7	27.7 ± 12.2	23.6 ± 10.5	24.4 ± 15.4	< 0.001
FIB-4 score	1.2 ± 0.8	1.2 ± 0.8	1.1 ± 0.8	1.2 ± 0.9	< 0.001
High-density lipoprotein cholesterol (mmol/L)	1.2 ± 0.3	1.3 ± 0.4	1.5 ± 0.4	1.4 ± 0.4	< 0.001
Total cholesterol (mmol/L)	103.8 ± 2.7	103.7 ± 2.8	104.3 ± 2.9	104.0 ± 3.2	< 0.001
Fasting triglycerides (mmol/L)	4.9 ± 1.1	4.9 ± 1.1	5.1 ± 1.1	5.2 ± 1.2	< 0.001
Comorbidities					
Hypertension	1116 (18.8%)	225 (18.8%)	1133 (17.0%)	159 (19.6%)	0.032
Diabetes	860 (14.5%)	184 (15.4%)	859 (12.9%)	172 (21.2%)	< 0.001
Cancer	558 (9.4%)	111 (9.3%)	607 (9.1%)	142 (17.5%)	< 0.001
CVD	339 (5.7%)	66 (5.5%)	142 (2.1%)	36 (4.5%)	< 0.001
Heart failure	202 (3.4%)	36 (3.0%)	152 (2.3%)	26 (3.2%)	0.002
Stroke	207 (3.5%)	39 (3.3%)	211 (3.2%)	54 (6.7%)	< 0.001
Emphysema/chronic bronchitis/both	89 (1.5%)/162 (2.7%)/47 (0.8%)	20 (1.7%)/56 (4.7%)/9 (0.8%)	28 (0.4%)/381 (5.7%)/30 (0.5%)	31 (3.9%)/102 (12.7%)/28 (3.5%)	< 0.001
Cigarettes per day	0.0 (0.0–40.0)	10.0 (0.0–95.0)	0.0 (0.0–16.0)	20.0 (15.0–95.0)	< 0.001
Alcohol consumption	0.1 (0.0–2.0)	1.0 (0.0–3.0)	0.0 (0.0–2.0)	0.0 (0.0–2.0)	< 0.001
Total physical activity*	360.0 (0.0–13,860.0)	1335.0 (0.0–14,760.0)	180.0 (0.0–10,500.0)	120.0 (0.0–10,560.0)	< 0.001
Leisure time physical activity*	120.0 (0.0–1380.0)	480.0 (0.0–8760.0)	75.0 (0.0–5880.0)	0.0 (0.0–6300.0)	< 0.001
HEI	52.3 ± 13.3	52.4 ± 12.6	55.4 ± 13.5	52.0 ± 13.5	< 0.001
Sedentary time (min/day)	360.0 (0.0–1080.0)	300.0 (4.0–9999.0)	300.0 (0.0–9999.0)	360.0 (0.0–1200.0)	< 0.001
Sleep hour	6.8 ± 1.4	6.9 ± 1.5	6.9 ± 1.4	6.8 ± 1.5	< 0.001

*NAFLD* non-alcoholic fatty liver disease, *BMI* body mass index, *FIB-4* fibrosis-4, *CVD* cardiovascular disease, *HEI* healthy eating index.

*Minutes per week.

### Association of lifestyle profiles with the risk of NAFLD and survival of NAFLD cases

Table 2 showed the unadjusted and adjusted results for the association between risk factors including lifestyle profile and NAFLD development. We found that compared to profile 1, profile 2 (OR 0.79; 95% CI 0.63–0.98; *P* = 0.042) and profile 3 (OR 0.83; 95% CI 0.73–0.96; *P* = 0.013) had lower incidence for NAFLD. In contrast, profile 4 showed similar NAFLD prevalence compared to profile 1 (OR 0.95; 95% CI 0.71–1.29; *P* = 0.756). Additionally, other variables such as age, race, marital status and education level were also found to be associated with NAFLD incidence (shown in Table 2 in detail).

**Table 2:** Associations of different profiles with NAFLD in un-adjusted and multivariate regression models.

Variables	Un-adjusted		Adjusted	
	OR (95% CI)	*P* value	OR (95% CI)	*P* value
Age, years (≥ 20, < 30 as reference)				
≥ 30, < 40	2.59 (2.07–3.23)	< 0.001	2.30 (1.81–2.93)	< 0.001
≥ 40, < 50	3.48 (2.77–4.38)	< 0.001	3.02 (2.32–3.92)	< 0.001
≥ 50, < 60	3.87 (3.09–4.85)	< 0.001	3.47 (2.71–4.46)	< 0.001
≥ 60	2.74 (2.19–3.43)	< 0.001	2.69 (2.02–3.56)	< 0.001
Race (Mexican American as reference)				
Other hispanic	0.61 (0.48–0.76)	< 0.001	0.62 (0.49–0.80)	< 0.001
Non-hispanic white	0.52 (0.43–0.62)	< 0.001	0.45 (0.36–0.58)	< 0.001
Non-hispanic black	0.65 (0.52–0.82)	< 0.001	0.69 (0.53–0.90)	0.009
The other	0.23 (0.17–0.31)	< 0.001	0.24 (0.18–0.33)	< 0.001
Education (Less than 9th grade as reference)				
9-11th grade	0.67 (0.49–0.91)	0.012	0.90 (0.66–1.23)	0.515
High school or equivalent	0.75 (0.56–1.00)	0.051	1.06 (0.78–1.45)	0.706
Some college	0.71 (0.52–0.97)	0.037	1.11 (0.80–1.54)	0.545
College or above	0.48 (0.35–0.65)	< 0.001	0.66 (0.47–0.92)	0.020
Marital status (married/cohabited as reference)				
Widowed	0.73 (0.57–0.95)	0.022	0.66 (0.49–0.88)	0.008
Divorced/separated	1.10 (0.88–1.37)	0.405	0.92 (0.73–1.16)	0.470
Unmarried	0.40 (0.34–0.47)	< 0.001	0.61 (0.51–0.74)	< 0.001
Poverty income ratio	1.02 (0.97–1.07)	0.442	1.00 (0.95–1.05)	0.912
Employment (employed as reference)	0.91 (0.80–1.03)	0.153	0.85 (0.73–1.01)	0.067
Insurance (insured as reference)	0.93 (0.78–1.09)	0.370	0.93 (0.78–1.11)	0.437
Profile (profile 1 as reference)				
Profile 2	0.80 (0.63–1.00)	0.052	0.79 (0.63–0.98)	**0.042**
Profile 3	0.83 (0.74–0.95)	0.006	0.83 (0.73–0.96)	**0.013**
Profile 4	1.10 (0.83–1.46)	0.499	0.95 (0.71–1.29)	0.756

Significant values are in bold.

*NAFLD* non-alcoholic fatty liver disease, *OR* odds ratio, *CI* confidential interval.

**Figure 2:**
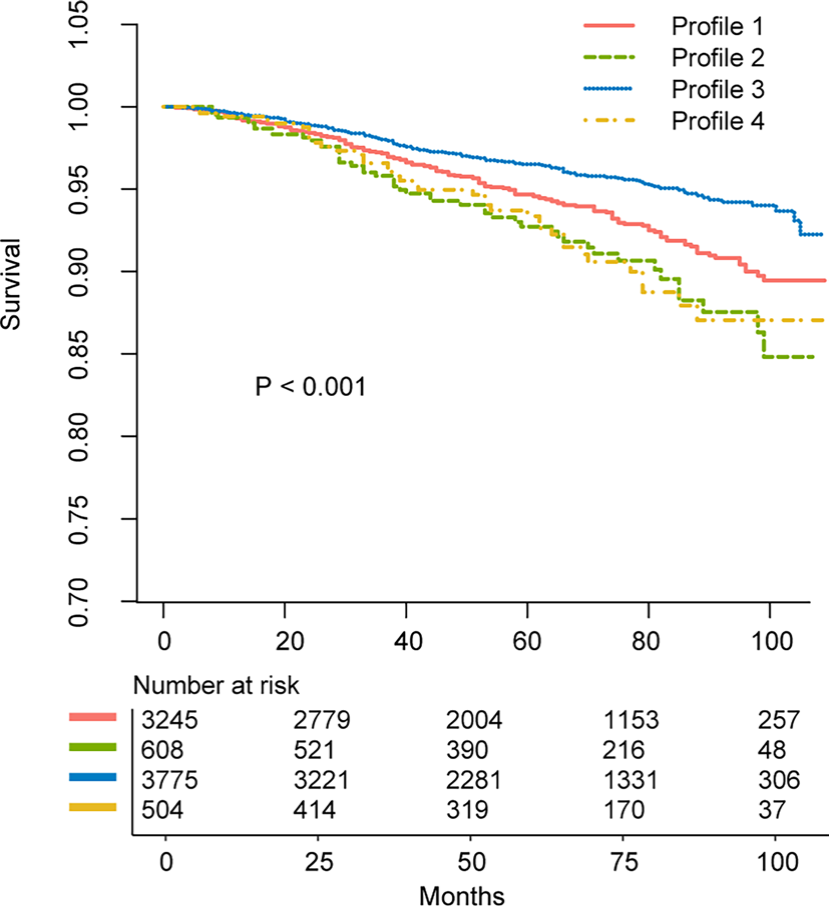
Unadjusted Kaplan–Meier survival curves for effect of lifestyle profile on all-cause mortality in patients with nonalcoholic fatty liver disease.

For the survival of NAFLD cases, individuals within profile 3 had the best long-term survival, and the HR was 0.55 (95% CI 0.40–0.76) for all-cause survival. Profile 4 (HR, 0.69; 95% CI 0.45–1.07; *P* = 0.098) and profile 2 (HR 1.14; 95% CI 0.74–1.75; *P* = 0.546) had similar survival compared to profile 1. In multivariate Cox regression, the following potential confounders were adjusted: age, race, education level, marital status, family income-to-poverty ratio, employment, insurance, BMI, FIB-4 score, and comorbidities including hypertension, diabetes, cancer, CVD, heart failure, stroke and COPD. The related HRs were presented in Table 3 in detail. In cases with NAFLD, the Kaplan–Meier curves also demonstrated that profile 3 had the best long-term survival (Fig. 2). In the total population (n = 14,622), cases within profile 3 also had better overall survival (Fig. S2A). Similar results were also observed in the population that only included those with HSI > 36 (with NAFLD) and < 30 (without NAFLD) (Fig. S2B).

**Table 3:** Associations of profiles with survival in participants with NAFLD in un-adjusted and adjusted models.

Variables	Un-adjusted		Adjusted	
	HR (95% CI)	*P* value	HR (95% CI)	*P* value
Age, years (≥ 20, < 30 as reference)				
≥ 30, < 40	0.93 (0.35–2.47)	0.890	0.94 (0.35–2.50)	0.898
≥ 40, < 50	2.17 (0.95–4.94)	0.065	2.00 (0.87–4.60)	0.102
≥ 50, < 60	2.57 (1.14–5.79)	0.023	2.07 (0.88–4.89)	0.096
≥ 60	10.16 (4.81–21.47)	< 0.001	3.78 (1.66–8.62)	0.002
Race (Mexican American as reference)				
Other hispanic	0.87 (0.53–1.44)	0.585	0.82 (0.48–1.39)	0.460
Non-hispanic white	1.46 (1.01–2.12)	0.047	1.17 (0.72–1.90)	0.533
Non-hispanic black	1.21 (0.81–1.83)	0.355	0.99 (0.55–1.79)	0.980
The other	0.69 (0.28–1.66)	0.402	0.71 (0.27–1.81)	0.468
Education (Less than 9th grade as reference)				
9–11th grade	0.63 (0.41–0.97)	0.035	0.88 (0.55–1.39)	0.578
High school or equivalent	0.46 (0.31–0.67)	< 0.001	0.76 (0.48–1.20)	0.241
Some college	0.47 (0.31–0.71)	< 0.001	0.97 (0.61–1.54)	0.887
College or above	0.35 (0.22–0.55)	< 0.001	0.97 (0.55–1.73)	0.928
Marital status (married/cohabited as reference)				
Widowed	5.84 (4.48–7.62)	< 0.001	2.14 (1.55–2.97)	< 0.001
Divorced/separated	1.52 (0.99–2.32)	0.055	1.38 (0.91–2.09)	0.126
Unmarried	0.57 (0.35–0.94)	0.026	1.06 (0.60–1.89)	0.843
Poverty income ratio	0.80 (0.74–0.86)	< 0.001	0.80 (0.73–0.88)	< 0.001
Employment (employed as reference)	2.12 (1.83–2.45)	< 0.001	1.30 (1.11–1.52)	0.001
Insurance (insured as reference)	0.62 (0.41–0.94)	0.025	0.93 (0.57–1.52)	0.775
BMI, kg/m^2^	1.02 (1.00–1.04)	0.053	1.00 (0.98–1.03)	0.640
FIB-4 score	1.54 (1.42–1.67)	< 0.001	1.26 (1.14–1.40)	< 0.001
Comorbidities (yes as reference)				
Hypertension	0.41 (0.31–0.54)	< 0.001	0.99 (0.75–1.29)	0.925
Diabetes	0.34 (0.26–0.46)	< 0.001	0.82 (0.62–1.09)	0.164
Cancer	0.32 (0.22–0.47)	< 0.001	0.64 (0.43–0.95)	0.028
CVD	0.20 (0.14–0.28)	< 0.001	0.71 (0.49–1.04)	0.079
Heart failure	0.11 (0.08–0.15)	< 0.001	0.43 (0.29–0.63)	< 0.001
Stroke	0.22 (0.16–0.29)	< 0.001	0.76 (0.55–1.05)	0.085
COPD	0.72 (0.66–0.80)	< 0.001	0.94 (0.86–1.04)	0.263
Profile (profile 1 as reference)				
Profile 2	1.50 (1.04–2.17)	0.030	1.14 (0.74–1.75)	0.546
Profile 3	0.76 (0.56–1.02)	0.067	0.55 (0.40–0.76)	< 0.001
Profile 4	1.42 (0.91–2.21)	0.125	0.69 (0.45–1.07)	0.098

*NAFLD* non-alcoholic fatty liver disease, *HR* hazard ratio, *CI* confidential interval, *BMI* body mass index, *FIB-4* Fibrosis-4, *CVD* cardiovascular disease, *COPD* chronic obstructive pulmonary disease.

### Associations of HLS with mortality and incident NAFLD by profiles

In Fig. S3A, we found a negative association between the number of healthy lifestyle factors and the prevalence of NAFLD. Additionally, the number of healthy lifestyle factors was also negatively associated with the long-term survival of participants with NAFLD. Furtherly, we explored the associations of healthy lifestyle score with mortality and incident NAFLD stratified by lifestyle profiles (Table 4). Interestingly, we observed that with the increase of HLS, participants within profile 2 did not show lower NAFLD incidence and better prognosis in NAFLD cases. For example, in profile 2, compared to those with 0–2 HLS, both of cases with 3–4 HLS (OR 0.72; 95% CI 0.47–1.08; *P* = 0.121) and 5–6 HLS (OR 0.73; 95% CI 0.33–1.60; *P* = 0.434) had similar incidence of NAFLD. For all-cause death, cases with 3–4 HLS (HR 0.72; 95% CI 0.39–1.32; *P* = 0.294) and 5–6 HLS (HR 0.70; 95% CI 0.21–2.39; *P* = 0.573) also had similar HRs in comparison to those with 0–2 HLS. In contrast, for the other three profiles, with the increase of HLS, individuals tended to have lower NAFLD incidence and better long-term survival. For example, in profile 1, cases with 3–4 HLS (OR, 0.79; 95% CI 0.66–0.94; *P* = 0.013) or 5–6 HLS (OR, 0.61; 95% CI 0.43–0.87; *P* = 0.009) had lower risk for NAFLD compared to those with 0–2 HLS. Cases with 5–6 HLS in profile 1 also displayed better survival compared to those with 0–2 HLS (HR 0.45; 95% CI 0.25–0.80; *P* = 0.007). In Fig. S4, we showed the unadjusted Kaplan–Meier analyses for effect of HLS on all-cause mortality in patients with NAFLD, separately by lifestyle profile.

**Table 4:** Associations of healthy lifestyle score with mortality and incident NAFLD by profiles.

Variables	0–2 HLS	3–4 HLS		5–6 HLS	
	Reference	OR/HR (95%CI)	*P* value	OR/HR (95%CI)	*P* value
NAFLD incidence					
Profile 1	1	0.79 (0.66–0.94)	0.013	0.61 (0.43–0.87)	0.009
Profile 2	1	0.72 (0.47–1.08)	0.121	0.73 (0.33–1.60)	0.434
Profile 3	1	0.93 (0.73–1.18)	0.542	0.65 (0.47–0.91)	0.014
Profile 4	1	0.51 (0.32–0.81)	0.007	0.96 (0.20–4.61)	0.960
All-cause death					
Profile 1	1	0.81 (0.60–1.11)	0.187	0.45 (0.25–0.80)	0.007
Profile 2	1	0.72 (0.39–1.32)	0.294	0.70 (0.21–2.39)	0.573
Profile 3	1	0.79 (0.56–1.10)	0.167	0.38 (0.24–0.59)	< 0.001
Profile 4	1	0.68 (0.39–1.19)	0.179	0.16 (0.03–0.96)	0.045

The following variables were adjusted: sex, age, race, education, marital status, family income to poverty ratio, employment, insurance.

*NAFLD* non-alcoholic fatty liver disease, *HLS* healthy lifestyle score, *OR* odds ratio, *HR* hazard ratio, *CI* confidential interval.

## Discussion

In this study, we identified multi-faceted lifestyle patterns in cases within NHANES 2007–2014 and investigated the associations between lifestyle profiles and the risk of NAFLD. Besides, in participants with NAFLD, we also examined the role of lifestyle profile in all-cause mortality. Four profiles were used to characterize the lifestyle patterns of the included participants. One of our main findings was that high adherence to the prudent lifestyle pattern (profile 3), characterized by high HEI score and lower cigarettes/alcohol consumptions, was significantly associated with lower odds of NAFLD. Profile 3 also showed the best long-term survival for cases with NAFLD. This association was independent of comorbidities, family sociodemographic-related and the other risk factors. Moreover, profile 2 (with high PA time, and high cigarettes/alcohol consumption) had lower risk for NAFLD than profile 1 (all lifestyle factors were in moderate levels). In this study, we observed that for cases with profile 2, lifestyle improvement could not correspondingly decrease the NAFLD incidence or improve the overall survival rate.

Given the differences of lifestyle in male and female, we constructed the lifestyle patterns for male and female separately^25^. In this study, significant differences were observed between male and female in lifestyle patterns. For example, the profile 2 in male showed both of the high total PA and LTPA time, but also had high cigarettes/alcohol consumptions, whereas the profile 3 in female presented with high HEI score, and moderate PA time. Interestingly, we found that profile 4 in female had the highest cigarettes consumption and lowest PA time (both total PA and LTPA). Consequently, profile 4 displayed higher NAFLD risk and worse prognosis after NAFLD. Based on the above results, a gender-specific approach to maintain healthy lifestyles among cases with high risk of NAFLD is highly recommended.

Owing to the favorable outcomes of profile 3, the role of diet quality in NAFLD development and prognosis should be strengthened and emphasized furtherly. Consistently, in the previous studies, Yoo, et al. demonstrated that high diet quality was inversely associated with the risk of NAFLD^14^. Zhang, et al. found that dietary patterns rich in animal foods or sugar were associated with a higher risk of NAFLD, while a vegetable rich dietary pattern was not^26^. Ivancovsky-Wajcman, et al. and Zhang, et al. showed that high ultra-processed food was associated with NAFLD^27,28^. Based on these findings, recommendation of high-quality diet may be an effective and beneficial goal for cases at risk for NAFLD. The protective role of physical activities and sedentary time were also clearly observed in existed literatures^29–32^. In the current study, the lower NAFLD risk in profile 2 compared to profile 1 may be predominantly attributed by the PA. Moreover, the low PA time in profile 4 may be associated with the worse outcomes in this profile. For cases in profile 4, one of the major improvements of lifestyle was the advocation of total and leisure time activities. As for smoking, widespread appeared in profiles 2 (male) and 4 (female), was also worthy of attention. More evidences have illustrated the harmful role of cigarette smoking in NAFLD development^15,33,34^. Previous studies showed that oxidative stress caused by smoking is a key mechanism underlying development and progression of NAFLD^35^. However, the detailed mechanisms between smoking and development of NAFLD should be furtherly explored and illustrated. Besides, the previous research demonstrated that a decrease in sleep duration or poor sleep quality over time was correlated with an increased risk of incident NAFLD^36^, which indicated that profiles with higher sleep hour (such as profile 2) may had better outcomes than those without enough sleep time.

For profile 2, the improvement of lifestyle did not bring better outcomes for participants correspondingly. This observation may be explained with the mechanism below. The effect of different lifestyle factors on NAFLD may be different. Cases within profile 2 already had high activity time and low sedentary time (the other lifestyle factors were also acceptable except for higher cigarette/alcohol consumption), thus, further change of the other lifestyles may be limited in overall outcome improvement. Consequently, the prevention of NAFLD development and improvement of overall survival for cases with profile 2 should not limited to lifestyle correction. Instead, the other risk factors should be changed or improved to avoid NAFLD development or decrease their deaths. For example, the improvement of socioeconomic status simultaneously of those at high risk of NAFLD may be effective to decrease NAFLD development^37,38^. In contrast, the improvement of lifestyle for the other profiles may be beneficial for preventing NAFLD occurrence and improve long-term prognosis for NAFLD patients. This was proved by evidences in the current study (the total number of healthy lifestyle factors was negatively correlated with NAFLD development and prognosis).

A strength of the current study lies in the selection of lifestyle factors. We identified seven continuous variables aimed to represent the lifestyle landscape of the participants. To our knowledge, no previous studies have investigated the association between a combination of lifestyle factors and NAFLD incidence. The categorization of lifestyle features derived from the LPA may have implication for case management and decision-making. It could help in planning management strategies tailored to subgroups of cases with different lifestyle tendencies. Additionally, the profiles in the current study allowed a better selection of candidates for NAFLD rehabilitation trials as well as fostering future studies on the pathophysiological mechanism of NAFLD development. However, several limitations in the current study should also be acknowledged. First, given the self-reported questionnaire used for the lifestyle or some other covariates such as comorbidities, some random misclassification errors in exposure assessment may exist. Second, it has been documented in the existing literature that diverse cancer types, occupational characteristics such as work type and duration, obstructive sleep apnea syndrome (OSAS), and various other factors potentially exhibit associations with NAFLD. However, upon meticulous examination of the NHANES data, we observed a lack of pertinent variables across different survey year cycles, which may resulting in a substantial reduction in sample size. As a consequence, the analysis outcomes may suffer from inadequate validity, leading us to refrain from incorporating these variables into our study.Third, even seven types of lifestyle-related variables were identified, it may not capture all lifestyle characteristics of the participants. Specially, in this study, the alcohol use was still used as a lifestyle factor in LPA analysis, and we found that in profile 2 the alcohol use was higher than the other profiles. Based on the NAFLD definition^20^, we only excluded those with significant alcohol consumption before analysis. However, it should be validated in the future whether all cases with alcohol drinking should be excluded when diagnosis of NAFLD. Moreover, the association of lifestyle and NAFLD incidence is based on the cross-sectional design and the causal relationship between lifestyle profile and NAFLD incidence cannot be evaluated directly because of the inability to assess temporal relationship with the NHANES data.

In conclusion, this study revealed that the lifestyles in different populations were heterogeneous, and cases could be classified into a typical subgroup based on the lifestyle factors. The data-driven lifestyle profile presented in this study was significantly associated with the risk of NAFLD and the survival of NAFLD cases. The lifestyle profile has the potential to improve lifestyle monitor plans for cases at high risk for NAFLD, and design management plans for a more personalized approach for rehabilitation of NAFLD.

### Supplementary Information

Supplementary Information.

## Data Availability

The National Health and Nutrition Examination Survey (NHANES) data is publicly available at https://www.cdc.gov/nchs/nhanes/index.htm.

## References

[1] Friedman SL, Neuschwander-Tetri BA, Rinella M, Sanyal AJ (2018). Mechanisms of NAFLD development and therapeutic strategies. Nat. Med..

[2] Powell EE, Wong VW, Rinella M (2021). Non-alcoholic fatty liver disease. Lancet (London, England)..

[3] Sheka AC, Adeyi O, Thompson J, Hameed B, Crawford PA, Ikramuddin S (2020). Nonalcoholic steatohepatitis: A review. JAMA..

[4] Loomba R, Friedman SL, Shulman GI (2021). Mechanisms and disease consequences of nonalcoholic fatty liver disease. Cell..

[5] Notarnicola, M., Osella, A. R., Caruso, M. G., et al. Nonalcoholic fatty liver disease: Focus on new biomarkers and lifestyle interventions. *Int. J. Mol. Sci.* 2021;**22**(8). 10.3390/ijms22083899 PMC8069944 33918878

[6] Prabhakar O, Bhuvaneswari M (2021). Role of diet and lifestyle modification in the management of nonalcoholic fatty liver disease and type 2 diabetes. Tzu chi Med J..

[7] El-Agroudy NN, Kurzbach A, Rodionov RN (2019). Are lifestyle therapies effective for NAFLD treatment?. Trends Endocrinol Metab: TEM..

[8] Parry SA, Turner MC, Hodson L (2020). Lifestyle interventions affecting hepatic fatty acid metabolism. Curr. Opin. Clin. Nutr. Metab. Care..

[9] Ahmed IA, Mikail MA, Mustafa MR, Ibrahim M, Othman R (2019). Lifestyle interventions for non-alcoholic fatty liver disease. Saudi J. Biol. Sci..

[10] Zou TT, Zhang C, Zhou YF (2018). Lifestyle interventions for patients with nonalcoholic fatty liver disease: A network meta-analysis. Eur. J. Gastroenterol. Hepatol..

[11] Hallsworth K, Adams LA (2019). Lifestyle modification in NAFLD/NASH: Facts and figures. JHEP Rep.: Innov. Hepatol..

[12] Vachliotis I, Goulas A, Papaioannidou P, Polyzos SA (2022). Nonalcoholic fatty liver disease: Lifestyle and quality of life. Hormones (Athens, Greece)..

[13] Chun, H. S., Lee, M., Lee, H. A., et al. Association of physical activity with risk of liver fibrosis, sarcopenia, and cardiovascular disease in nonalcoholic fatty liver disease. *Clin. Gastroenterol. Hepatol.* 2022. 10.1016/j.cgh.2021.12.043 34998993

[14] Yoo ER, Kim D, Vazquez-Montesino LM (2020). Diet quality and its association with nonalcoholic fatty liver disease and all-cause and cause-specific mortality. Liver Int.: Official J. Int. Assoc. Study Liver..

[15] Fouda, S., Khan, A., Chan, S. M. H., et al. Exposure to cigarette smoke precipitates simple hepatosteatosis to NASH in high-fat diet fed mice by inducing oxidative stress. *Clin. Sci. (London, England: 1979).* 2021;**135**(17):2103–2119. 10.1042/CS20210628 PMC8436265 34427662

[16] Eguchi E, Iso H, Tanabe N (2012). Healthy lifestyle behaviours and cardiovascular mortality among Japanese men and women: the Japan collaborative cohort study. Eur. Heart J..

[17] Vajdi M, Nikniaz L, Pour Asl AM, Abbasalizad FM (2020). Lifestyle patterns and their nutritional, socio-demographic and psychological determinants in a community-based study: A mixed approach of latent class and factor analyses. PloS one..

[18] Davis JS, Banfield E, Lee HY, Peng HL, Chang S, Wood AC (2019). Lifestyle behavior patterns and mortality among adults in the NHANES 1988–1994 population: A latent profile analysis. Preventive medicine..

[19] Hajifathalian K, Torabi Sagvand B, McCullough AJ (2019). Effect of alcohol consumption on survival in nonalcoholic fatty liver disease: A national prospective cohort study. Hepatology (Baltimore, Md).

[20] Kim D, Vazquez-Montesino LM, Li AA, Cholankeril G, Ahmed A (2020). Inadequate physical activity and sedentary behavior are independent predictors of nonalcoholic fatty liver disease. Hepatol. (Baltimore, Md.).

[21] Zhang YB, Chen C, Pan XF (2021). Associations of healthy lifestyle and socioeconomic status with mortality and incident cardiovascular disease: two prospective cohort studies. BMJ (Clin Res ed.).

[22] Wang, K., Zhao, Y., Nie, J., Xu, H., Yu, C., Wang, S. Higher HEI-2015 score is associated with reduced risk of depression: Result from NHANES 2005–2016. *Nutrients.* **13**(2) (2021). 10.3390/nu13020348 PMC7911826 33503826

[23] Kim D, Li AA, Ahmed A (2018). Leucocyte telomere shortening is associated with nonalcoholic fatty liver disease-related advanced fibrosis. Liver Int..

[24] Bauer GR, Mahendran M, Walwyn C, Shokoohi M (2022). Latent variable and clustering methods in intersectionality research: Systematic review of methods applications. Soc. Psychiatry Psychiatric Epidemiol..

[25] Yoshioka N, Ishigami M, Watanabe Y (2020). Effect of weight change and lifestyle modifications on the development or remission of nonalcoholic fatty liver disease: Sex-specific analysis. Sci. Rep..

[26] Zhang S, Gu Y, Bian S (2021). Dietary patterns and risk of non-alcoholic fatty liver disease in adults: A prospective cohort study. Clin. Nutr. (Edinburgh, Scotland)..

[27] Ivancovsky-Wajcman D, Fliss-Isakov N, Webb M (2021). Ultra-processed food is associated with features of metabolic syndrome and non-alcoholic fatty liver disease. Liver Int..

[28] Zhang S, Gan S, Zhang Q (2022). Ultra-processed food consumption and the risk of non-alcoholic fatty liver disease in the Tianjin Chronic Low-grade Systemic Inflammation and Health Cohort Study. Int. J. Epidemiol..

[29] Berzigotti A, Saran U, Dufour JF (2016). Physical activity and liver diseases. Hepatology (Baltimore, Md.).

[30] Kim, D., Konyn, P., Cholankeri, l. G., Ahmed, A. Physical activity is associated with nonalcoholic fatty liver disease and significant fibrosis measured by fibroscan. *Clin. Gastroenterol. Hepatol.* (2021). 10.1016/j.cgh.2021.06.029 34214678

[31] Kim D, Murag S, Cholankeril G (2021). Physical activity, measured objectively, is associated with lower mortality in patients with nonalcoholic fatty liver disease. Clin Gastroenterol Hepatol.

[32] Kerr J, Anderson C, Lippman SM (2017). Physical activity, sedentary behaviour, diet, and cancer: An update and emerging new evidence. Lancet. Oncol..

[33] Okamoto M, Miyake T, Kitai K (2018). Cigarette smoking is a risk factor for the onset of fatty liver disease in nondrinkers: A longitudinal cohort study. PloS one..

[34] Hamabe A, Uto H, Imamura Y (2011). Impact of cigarette smoking on onset of nonalcoholic fatty liver disease over a 10-year period. J. Gastroenterol..

[35] Rinaldi, L., Pafundi, P. C., Galiero, R., et al. Mechanisms of non-alcoholic fatty liver disease in the metabolic syndrome. A narrative review. *Antioxidants (Basel, Switzerland).* **10**(2) (2021). 10.3390/antiox10020270 PMC7916383 33578702

[36] Um, Y. J., Chang, Y., Jung, H. S., et al. Decrease in sleep duration and poor sleep quality over time is associated with an increased risk of incident non-alcoholic fatty liver disease. *J. Personal. Med.* **12**(1) (2022). 10.3390/jpm12010092 PMC8777783 35055407

[37] Vilar-Gomez, E., Nephew, L. D., Vuppalanchi, R., *et al*. High-quality diet, physical activity, and college education are associated with low risk of NAFLD among the US population. *Hepatology (Baltimore, Md.)* (2021). 10.1002/hep.32207 34668597

[38] Cho, J., Lee, I., Park, D. H., Kwak, H. B., Min, K. Relationships between socioeconomic status, handgrip strength, and non-alcoholic fatty liver disease in middle-aged adults. *Int. J. Environ. Res. Public Health.* **18**(4) (2021). 10.3390/ijerph18041892 PMC7920055 33669288

