# Supplementary material for: Optimizing lifestyle profiles is potential for preventing nonalcoholic fatty liver disease and enhancing its survival
Source: Sci Rep. 2024 Mar 6;14:5516. doi: 10.1038/s41598-024-55566-9 (PMC10918176; doi:10.1038/s41598-024-55566-9)
Supplement: Supplementary file 1 — Supplementary Information. [file 41598_2024_55566_MOESM1_ESM.docx]

**Optimizing Lifestyle Profiles is potential for Preventing Nonalcoholic Fatty Liver Disease and Enhancing its Survival**

Beilin Tu^1^, Wei Li^1^, Haitao Xiao^1^, Xuewen Xu^1^, Yange Zhang^1,2^

^1^Department of Plastic and Burns Surgery, West China Hospital, Sichuan University, Chengdu 610041, China;

^2^Department of Liver Surgery and Liver Transplantation, State Key Laboratory of Biotherapy and Cancer Center, West China Hospital, Sichuan University and Collaborative Innovation Center of Biotherapy, Chengdu 610041, China

Correspondence: Department of Plastic and Burns Surgery, West China Hospital, Sichuan University, 37 Guoxue Road, Chengdu 610041, China. E-mail address: zhangyange7801@163.com (Y. Zhang); xxw_0826@163.com (X. Xu)..

| Table S1 Latent profile analysis for determining the best number of profiles | | | | | | | | | |  |
| --- | --- | --- | --- | --- | --- | --- | --- | --- | --- | --- |
| Number of classes | AIC | CAIC | BIC | SABIC | Entropy | Number of patients in each latent profile | | | |  |
|  |  |  |  |  |  | 1 | 2 | 3 | 4 |  |
| Male |  |  |  |  |  |  |  |  |  |  |
| 2 | 171376 | 171531 | 171512 | 171452 | 0.958 | 7718 (79.4%) | 2000 (20.6%) |  |  |  |
| 3 | 171303 | 171516 | 171490 | 171407 | 0.584 | 6082 (62.6%) | 2952 (30.4%) | 684 (7.0%) |  |  |
| 4 | 166516 | 166786 | 166753 | 166648 | 0.663 | 5952 (61.2%) | 2650 (27.3%) | 678 (7.0%) | 438 (4.5%) |  |
| Female |  |  |  |  |  |  |  |  |  |  |
| 2 | 146846 | 147002 | 146983 | 146922 | 0.965 | 8913 (89.3%) | 1067 (10.7%) |  |  |  |
| 3 | 134424 | 134638 | 134612 | 134529 | 0.546 | 5070 (50.8%) | 4889 (49.0%) | 21 (0.2%) |  |  |
| 4 | 146696 | 146967 | 146934 | 146829 | 0.478 | 4084 (40.9%) | 3579 (35.9%) | 1234 (12.4%) | 1086 (10.9%) |  |
| AIC, Akaike information criterion; CAIC, consistent Akaike information criterion; BIC, Bayesian information criteria; SABIC, sample-size adjusted Bayesian Information Criterion. | | | | | | | | | |  |
|  |  |  |  |  |  |  |  |  |  |  |

| Table S2 Associations of different profiles with NAFLD after psoriasis was also included in adjusted and multivariate regression models | | |
| --- | --- | --- |
| Variables | Adjusted | |
|  | OR (95% CI) | P value |
| Age, years (≥20, <30 as reference) |  |  |
| ≥30, <40 | 2.61 (2.14-3.18) | <0.001 |
| ≥40, <50 | 3.33 (2.68-4.13) | <0.001 |
| ≥50, <60 | 4.14 (3.27-5.24) | <0.001 |
| ≥60 | 2.81 (2.27-3.48) | <0.001 |
| Race (Mexican American as reference) |  |  |
| Other Hispanic | 0.60 (0.45-0.81) | <0.001 |
| Non-Hispanic White | 0.42 (0.33-0.53) | <0.001 |
| Non-Hispanic Black | 0.62 (0.48-0.80) | <0.001 |
| The other | 0.20 (0.15-0.26) | <0.001 |
| Education (Less than 9th grade as reference) |  |  |
| 9-11th grade | 0.76 (0.55-1.05) | 0.101 |
| High school or equivalent | 0.82 (0.60-1.11) | 0.201 |
| Some college | 0.87 (0.64-1.18) | 0.362 |
| College or above | 0.49 (0.36-0.68) | <0.001 |
| Marital status (married/cohabited as reference) |  |  |
| Widowed | 0.61 (0.47-0.79) | <0.001 |
| Divorced/separated | 0.82 (0.67-1.00) | 0.051 |
| Unmarried | 0.65 (0.55-0.77) | <0.001 |
| Poverty income ratio | 1.01 (0.96-1.05) | 0.745 |
| Employment (employed as reference) | 0.87 (0.76-1.01) | 0.061 |
| Insurance (insured as reference) | 1.02 (0.86-1.19) | 0.857 |
| Psoriasis (had psoriasis as reference) | 1.05 (0.73-1.50) | 0.797 |
| Profile (profile 1 as reference) |  |  |
| Profile 2 | 0.82 (0.66-1.03) | 0.093 |
| Profile 3 | 1.11 (0.97-1.27) | 0.143 |
| Profile 4 | 1.35 (1.00-1.82) | 0.048 |
| NAFLD, non-alcoholic fatty liver disease; OR, odds ratio; CI, confidential interval. | | |


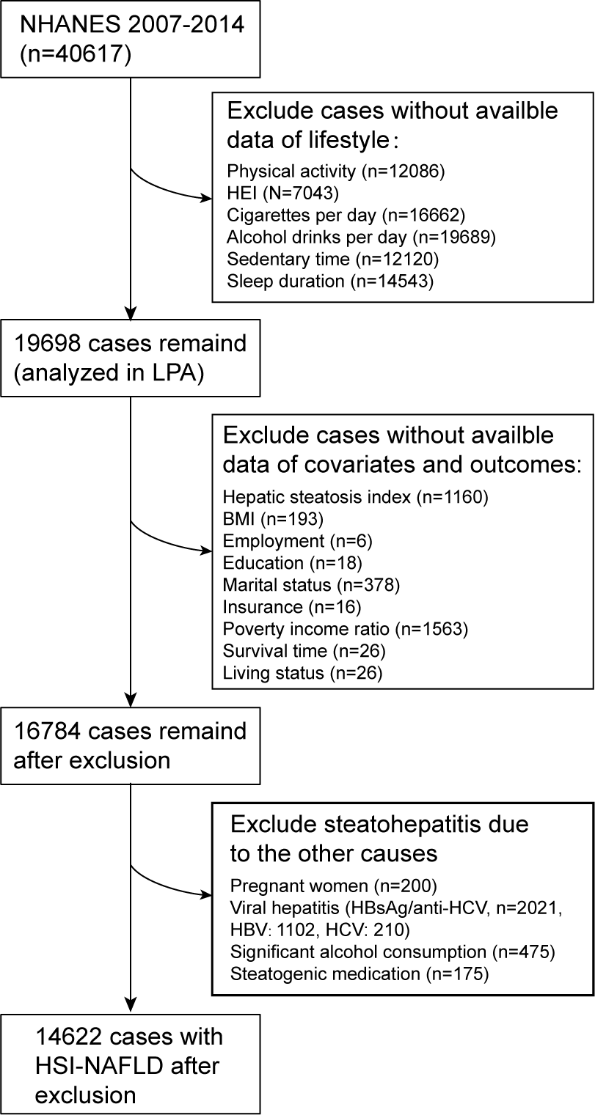


Fig. S1 Flowchart of the inclusion and exclusion criteria in the current study. NHANES, National Health and Nutrition Examination Survey; LPA, latent profile analysis; HSI, hepatic steatosis index; NAFLD, non-alcoholic fatty liver disease; HEI, healthy eating index; BMI, body mass index; HCV, hepatitis C; HBV, hepatitis B.


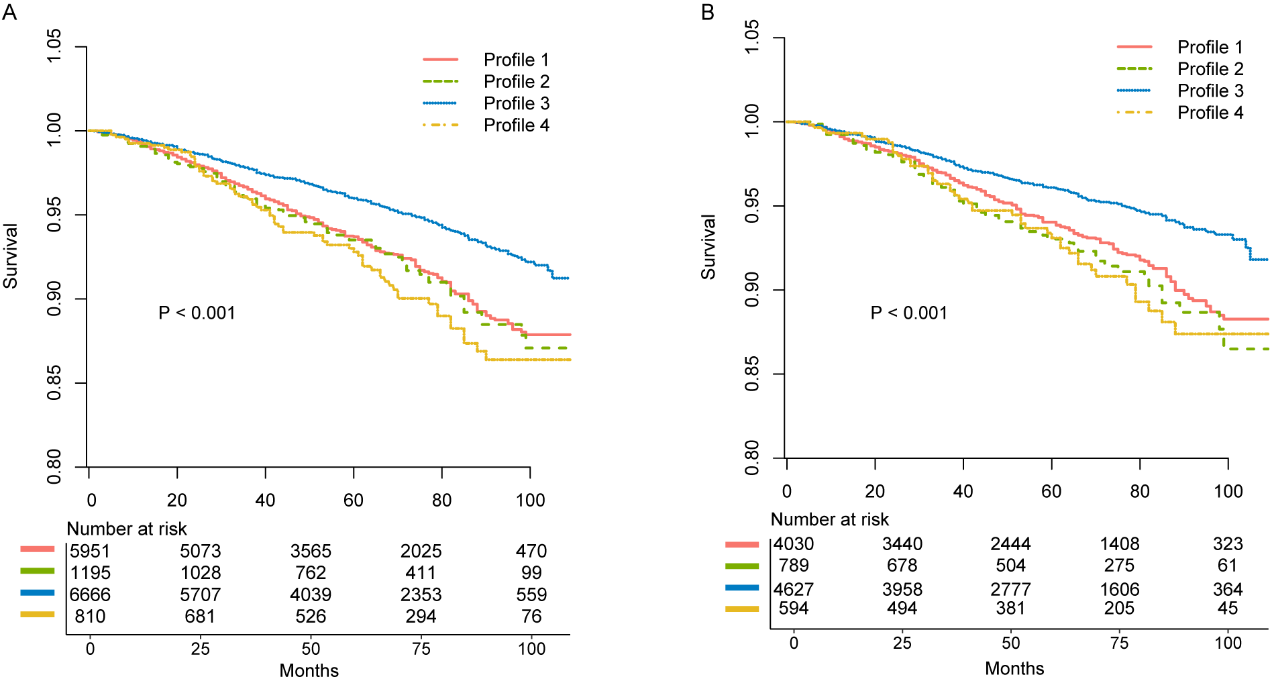


Fig. S2 Unadjusted Kaplan-Meier survival curves for effect of lifestyle profile on all-cause mortality in all participants (A) or those with HSI >36 or HSI <30 (B).


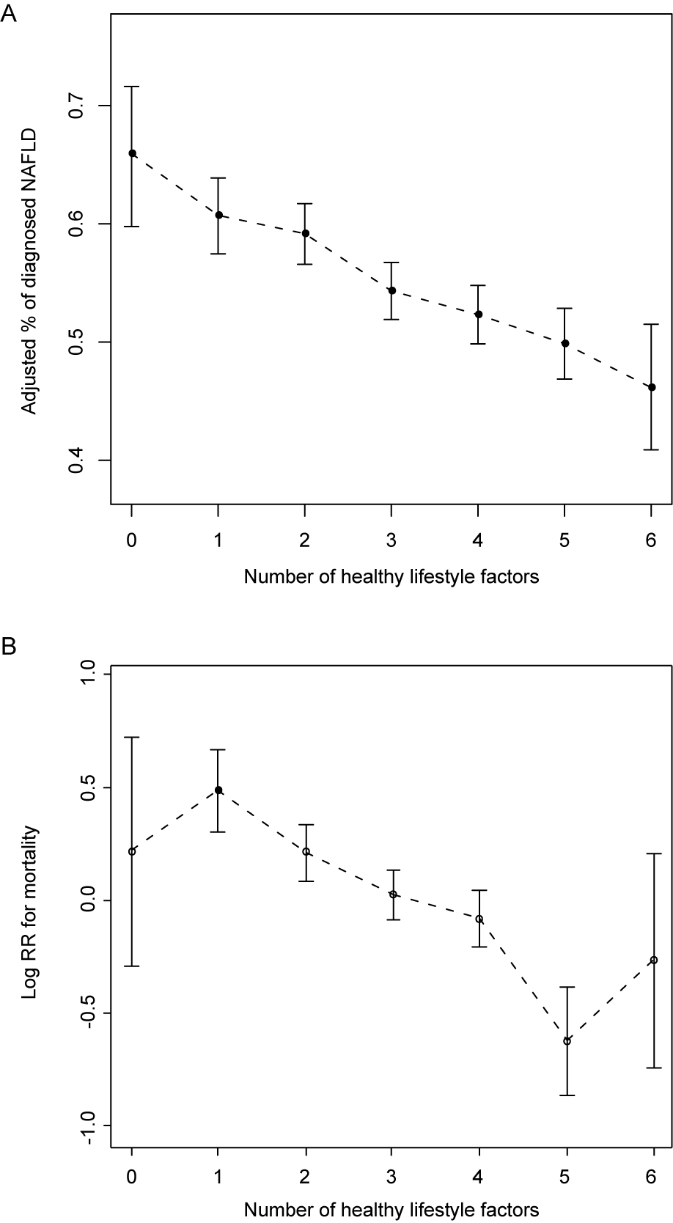


Fig. S3 Associations of the number of healthy lifestyle factors (healthy lifestyle score) with incident NAFLD (A) and all-cause survival (B). The following variables were adjusted: sex, age, race, education, marital status, family income to poverty ratio, employment, insurance. RR, risk ratio.


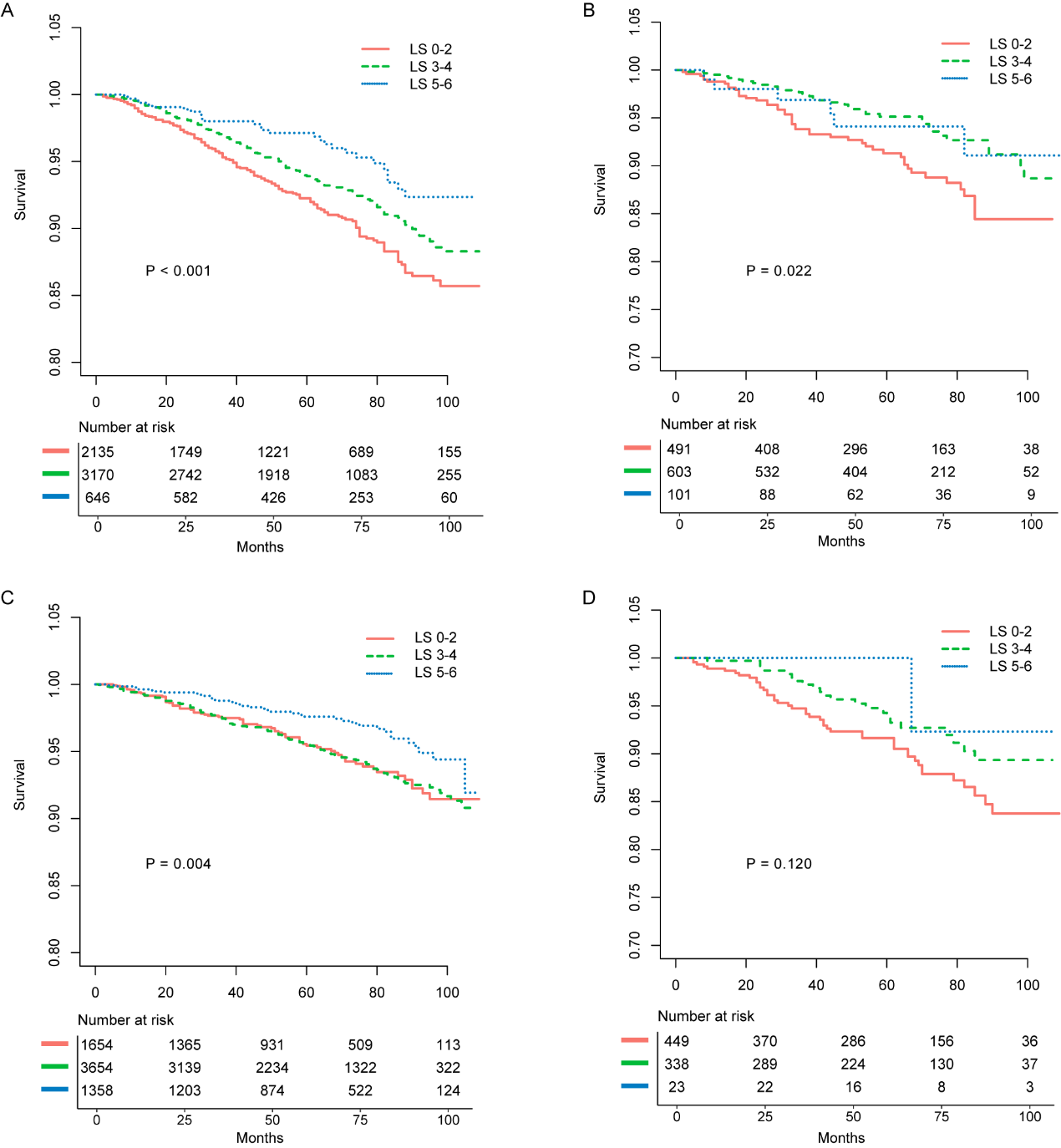


Fig. S4 Unadjusted Kaplan-Meier survival curves for effect of the number of healthy lifestyle factors (healthy lifestyle score) on all-cause mortality in patients with NAFLD, separately by profiles (A-D: profile 1-profile 4).
